# A General Strategy for Ordered Carrier Transport of Quasi-2D and 3D Perovskite Films for Giant Self-Powered Photoresponse and Ultrahigh Stability

**DOI:** 10.1007/s40820-023-01087-5

**Published:** 2023-04-30

**Authors:** Fei Zhu, Gang Lian, Deliang Cui, Qilong Wang, Haohai Yu, Huaijin Zhang, Qingbo Meng, Ching-Ping Wong

**Affiliations:** 1 State Key Laboratory of Crystal Materials, Shandong University, Jinan, 250100 People’s Republic of China; 2 Key Laboratory for Special Functional Aggregated Materials of Education Ministry, School of Chemistry & Chemical Engineering, Shandong University, Jinan, 250100 People’s Republic of China; 3 Key Laboratory for Renewable Energy, Chinese Academy of Sciences (CAS), Beijing, People’s Republic of China; 4 Key Laboratory for New Energy Materials and Devices, Beijing National Laboratory for Condensed Matter Physics, Institute of Physics, CAS, Beijing, 100190 People’s Republic of China; 5 School of Materials Science and Engineering, Georgia Institute of Technology, Atlanta, GA 30332 USA

**Keywords:** Perovskites, Thermal-pressed strategy, Uniaxial orientation, Self-powered photodetectors, Stability

## Abstract

Organic–inorganic hybrid perovskite materials have been focusing more attention in the field of self-powered photodetectors due to their superb photoelectric properties. However, a universal growth approach is required and challenging to realize vertically oriented growth and grain boundary fusion of 2D and 3D perovskite grains to promote ordered carrier transport, which determines superior photoresponse and high stability. Herein, a general thermal-pressed (TP) strategy is designed to solve the above issues, achieving uniaxial orientation and single-grain penetration along the film thickness direction. It constructs the efficient channel for ordered carrier transport between two electrodes. Combining of the improved crystal quality and lower trap-state density, the quasi-2D and 3D perovskite-based self-powered photodetector devices (with/without hole transport layer) all exhibit giant and stable photoresponse in a wide spectrum range and specific wavelength laser. For the MAPbI_3_-based self-powered photodetectors, the largest *R*_*λ*_ value is as high as 0.57 A W^−1^ at 760 nm, which is larger than most reported results. Meanwhile, under laser illumination (532 nm), the FPEA_2_MA_4_Pb_5_I_16_-based device exhibits a high responsivity (0.4 A W^−1^) value, which is one of the best results in 2DRP self-powered photodetectors. In addition, fast response, ultralow detection limit, and markedly improved humidity, optical and heat stabilities are clearly demonstrated for these TP-based devices.

**Supplementary Information:**

The online version contains supplementary material available at 10.1007/s40820-023-01087-5.

## Highlights

- Uniaxial orientation and single-grain penetration of quasi-2D and 3D perovskite films were achieved via a general thermal-pressed (TP) strategy.
- The TP-perovskite-based self-powered photodetectors (SPPDs) exhibit one of the best results among the 3D and quasi-2D perovskite SPPDs, respectively.
- These unpackaged TP-based devices exhibit ultrahigh humidity, optical and heat stability.

**Supplementary Information:**

The online version contains supplementary material available at 10.1007/s40820-023-01087-5.

## Graphical abstract

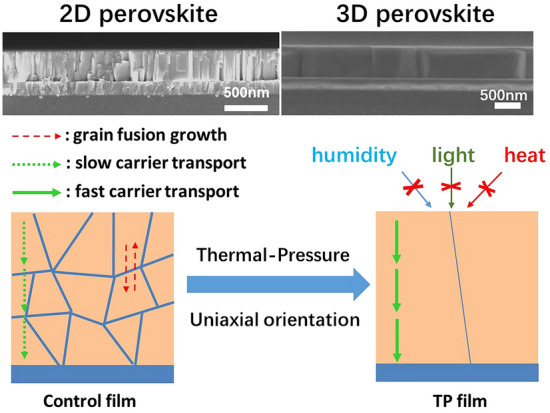

## Introduction

Nowadays, rapid growth of the Internet of Things has aroused strong demand for low-cost, high-performance photodetectors, which can be deployed in optical communication, image sensing, security, biological detection and intelligent monitoring [1, 2]. The ideal detector nodes should be requested to work with ultra-low detection limitation in self-powered mode [3], which relies on a built-in electric field generated by Schottky contacts or heterojunctions. Separation of photo-generated electron–hole pairs gives rise to a circuit current, which certainly requires active materials with excellent photoresponse behavior.

Organic–inorganic hybrid perovskite materials have been extensively studied in the field of self-powered photodetectors (SPPDs) due to their superb photoelectric properties, including large optical absorption coefficient, adjustable bandgap and long exciton diffusion length [4–6]. For example, Yang’s group reported a CH_3_NH_3_PbI_3-x_Cl_x_-based inverted device [7]. The performance is comparable to or even better than photodetectors fabricated from vacuum-processed inorganic materials. A MAPbI_3_-based self-driven narrowband photodetector with tunable spectral response was designed on the basis of the charge collection narrowing mechanism [8]. Zeng’s group reported bionic detectors based on low-bandgap inorganic perovskite, which was applied for selective NIR-I photon detection and imaging [9]. Despite the stunning development in three-dimensional (3D) perovskite-based SPPDs with high responsivity [10–12], the intrinsic issue of disordered microcrystal active layers, derived from rapid crystallization of precursor films, easily results in relatively poor thermal and moisture stability [13, 14]. Meanwhile, the abundant intragrain and interface defects are believed a major cause of carrier recombination, negatively affecting the photoresponse behavior. Recently, 2D Ruddlesden-Popper (2DRP) perovskites ((RNH_3_)_2_(A)_*n*-1_M_*n*_X_3*n*+1_) have come to the fore to promisingly achieve the balance between performance and stability owing to attractive quantum-well structure, flexibly tunable bandgap, and improved long-term stability compared with 3D counterparts [15–18]. Unfortunately, owing to habitual growth of 2DRP perovskites along in-plane direction, these insulating ligands (RNH_3_) restrain interlayer charge transport and inefficient charge collection. Thus, these improvements in stability have commonly been accompanied by degraded photoelectrical performance of photovaoltaic SPPDs [19–21].

Apparently, whether 3D or quasi-2D perovskite-based SPPDs, it is required to minimize the energy loss when the carriers transport through the active layers. For accelerating carriers transport and collection along the electric field direction in photovoltaic SPPDs, the achievement of uniaxial orientation of perovskite grains against the substrates can be an expectant solution to greatly enhance the photoelectrical performance of the devices. Here, the uniaxial orientation is defined as the crystals with the same out-of-plane orientation and without grain boundaries (GBs) in the film-thickness direction. In such case, the single-crystal channel with low intragrain and structural defects is constructed, which is favorable for photogenerated-carriers transport and electron–hole separation between two electrodes [22]. With the purpose of tuning the nucleation and crystallization process of perovskite thin films, some approaches have been developed, such as solvent-assisted annealing [23, 24] and additive engineering [25, 26]. Despite the remarkable improvement in film quality, the closely dependent relationship of specific strategy and perovskite system greatly limits their development. Facing a variety of 3D and 2D perovskite films with different composition and structures, it is still a tough challenge to prepare a series of uniaxial-orientation perovskite thin films via a general strategy.

In terms of grain-boundary fusion-growth thought, through introducing a key driving force of thermal-pressed (TP) effect in the spin-coated precursor films, several kinds of perovskite films with uniaxial orientation, including typical 3D CH_3_NH_3_PbI_3_ (MAPbI_3_), and quasi-2D (C_6_H_5_CH_2_CH_2_NH_3_)_2_(CH_3_NH_3_)_4_Pb_5_I_16_ (PEA_2_MA_4_Pb_5_I_16_) and 4-F-C_6_H_4_CH_2_CH_2_NH_3_)_2_(CH_3_NH_3_)_4_Pb_5_I_16_ (FPEA_2_MA_4_Pb_5_I_16_), are prepared directly on ITO glass and transport layers, respectively. The optimized thin films all present ultra-high smoothness close to that of single-grain one. The perovskite grains are uniformly oriented growth and vertical span in the entire film thickness, generating markedly lowered trap-state density and longer carrier lifetime. Subsequently, they present markedly enhanced responsivity (*R*_*λ*_) value under illumination of white light. For the MAPbI_3_-based SPPDs, the largest *R*_*λ*_ value is as high as 0.57 A W^−1^ at 760 nm, which is larger than most reported results. Meanwhile, under laser illumination (532 nm), the FPEA_2_MA_4_Pb_5_I_16_-based devices exhibit high responsivity (*R*, 0.4 A W^−1^) value, which is one of the best results in 2DRP perovskite-based SPPDs. In addition, fast response time, and enhanced stability are also clearly demonstrated for these SPPDs.

## Experimental Section

### Materials

Methyl-ammonium iodide (MAI), phenylethyl-ammonium iodide (PEAI) and 4-Fluoro-phenylethyl-ammonium iodide (FPEAI) were offered from Greatcell Solar Materials Pty Ltd. (NH_4_SCN) was offered from Sigma-Aldrich. Bahocuproine (BCP) was ordered from J&K, PbI_2_ was offered from TCI. Chlorobenzene (CB) and isopropyl alcohol (IPA), N, N-dimethylformamide (DMF) and N, N-dimethylsulfoxide (DMSO) were offered from Alfa Aesar. Silver was purchased from 3AChem. (6,6)-Phenyl-C61-butyric acid methyl ester (PC61BM) was offered from Lumtec. Poly (3,4-ethylenedioxythiophene): poly-(styrenesulfonate) (PEDOT:PSS) aqueous solution (Al 4083), Bphen were offered from Xi’an Polymer Light Technology Corporation.

### Fabrication of Perovskite Films and Devices

#### ***Fabrication of PEA***_***2***_***MA***_***4***_***Pb***_***5***_***I***_***16***_*** Perovskite Films and Devices***

The control film: first, clean the ITO glass ultrasonically with deionized water and ethanol, irradiate it with a UV lamp for four minutes, and then dry it with nitrogen. PEAI, MAI and PbI_2_ were mixed in 1 ml DMF at a molar ratio of 2:4:5 to prepare a precursor solution of perovskite, which was stirred at 70 °C overnight. The precursor solution was spin-coated with 5,000 rpm for 45 s to prepare the precursor film. Then, when it was annealed under 100 °C for 15 min. The obtained film was named as control film.

The SCN film: NH_4_SCN, PEAI, MAI and PbI_2_ are at a molar ratio of 2:2:4:5 to prepare a precursor solution of perovskite. The precursor solution was spin-coated with 5,000 rpm for 45 s to prepare the precursor film. Then, when it was annealed under 100 °C for 15 min. The obtained film was named as SCN film.

The SCN-TP film: the precursor SCN film is covered with a silicon wafer with good hydrophobicity, and then wrapped with a polytetrafluoroethylene film. Then it is transferred to a hot autoclave filled with simethicone, and the temperature and pressure of the film are given. During the pressure–thermal process, the pressure applied to the autoclave is 200 MPa, and it is maintained at all time. The temperature is first raised from 30 to 150 °C, kept for 12 h, and then cooled to 30 °C at a rate of 1 °C min^−1^. The prepared perovskite film is named as SCN-TP film.

The preparation of perovskite device: PCBM was deposited on the prepared perovskite film at a speed of 3,000 rpm for 30 s. Subsequently, BCP was spin-coated at 5,000 rpm for 30 s. Finally, 100 nm Ag is deposited on the top layer. The photodetector area is 0.04 cm^2^ with the metal mask.

#### ***Fabrication of FPEA***_***2***_***MA***_***4***_***Pb***_***5***_***I***_***16***_*** Perovskite Films and Devices***

The FPEA-control film: clean the ITO glass ultrasonically with deionized water and ethanol, irradiate it with a UV lamp for four minutes, and then dry it with nitrogen. PEDOT: PSS layer is spin-coated with 6000 rpm for 30 s, and annealed in an air environment at 150 °C for 15 min. NH_4_SCN, FPEAI, MAI and PbI_2_ are mixed in 1 mL DMF and 30 μL DMSO at a molar ratio of 2:2:4:5 to prepare a precursor solution of perovskite, which was stirred at 70 °C overnight. The precursor solution is spin-coated with 5,000 rpm for 45 s to prepare the precursor film. Then, when it is annealed under 100 °C for 15 min. The obtained film was named as FPEA-control film.

The FPEA-TP film: During the pressure–thermal process, the pressure applied to the autoclave is 200 MPa, and it is maintained at all time. The temperature is first raised from 30 to 150 °C, kept for 12 h, and then cooled to 30 °C at a rate of 1 °C min^−1^. The prepared perovskite film is named as FPEA-TP film.

The preparation of perovskite device: PCBM was deposited on the prepared perovskite film at a speed of 3,000 rpm for 30 s. Subsequently, BCP was spin-coated at 5,000 rpm for 30 s. Finally, 100 nm Ag is deposited on the top layer. The photodetector area is 0.04 cm^2^ with the metal mask.

#### ***Fabrication of MAPbI***_***3***_*** Perovskite Films and Devices***

The MA-control film: clean the ITO glass ultrasonically with deionized water and ethanol, irradiate it with a UV lamp for four minutes, and then dry it with nitrogen. PEDOT: PSS layer is spin-coated with 6,000 rpm for 30 s, and annealed in an air environment at 120 °C for 10 min. MAI and PbI_2_ are mixed in 1 mL of DMF/DMSO (volume ratio, 9:1) at a molar ratio of 1.1:1 to prepare a precursor solution of perovskite and a concentration of 2 M, which was stirred at 70 °C overnight. The precursor solution is spin-coated with 6,000 rpm for 30 s, after the 10 s, 300 μL *sec*-butanol were added. Then, the film was pre-annealed at 75 °C for 10 min. The obtained film was named as FPEA-control film.

The MA-TP film: During the pressure–thermal process, the pressure applied to the autoclave is 150 MPa, and it is maintained at all time. The temperature is first raised from 30 to 100 °C, kept for 5 h, and then cooled to 30 °C at a rate of 0.058 °C min^−1^. The prepared perovskite film is named as FPEA-TP film.

The preparation of perovskite device: PC_61_BM was deposited on the prepared perovskite film at a speed of 3,000 rpm for 30 s. Subsequently, Bphen was spin-coated at 6,000 rpm for 30 s. Finally, 100 nm Ag is deposited on the top layer. The photodetector area is 0.04 cm^2^ with the metal mask.

### Measurements and Characterization

High resolution X-ray diffractometer (XRD) patterns were acquired on a SmartLab3 KW diffractometer. ZEISS G300 emission scanning electron microscope was performed for SEM images. Ultraviolet-2600 spectrophotometer recorded the ultraviolet–visible absorption spectrum. AFM was measured by Dimensional Icon atomic force microscope. The impedance analyzer (CHI 660E) was used to characterize the electrochemical impedance spectroscopy (EIS) under dark conditions. 532 nm laser diode is employed as the light source of photodetector, and the incident irradiation power is surveyed by power meter (PM400, thorlabs). The noise measurement system (DA platform, NC 300A) records the noise power spectrum from 1 Hz to 100 kHz. For *SCLC* measurement, the dark current–voltage (*I-V*) curves were measured on a Keithley 4200-SCS semiconductor parameter analyzer. Photoelectric features of the 2DRP film photodetectors were recorded on a Keithley 4200-SCS semiconductor parameters analyzer at room temperature in air. The GIWAXS measurements were performed of Xeuss 2.0 using X-ray with a wavelength of 1.54189. The polar diagram was characterized by PANalytical X'Pert^3^ MRD. The external quantum efficiency (EQE) was tested employing an EQE system (Enlitech QE-R3011) equipped with a xenon lamp.

## Results and Discussion

### Structural Characterization of Perovskite Films

**Fig. 1:**
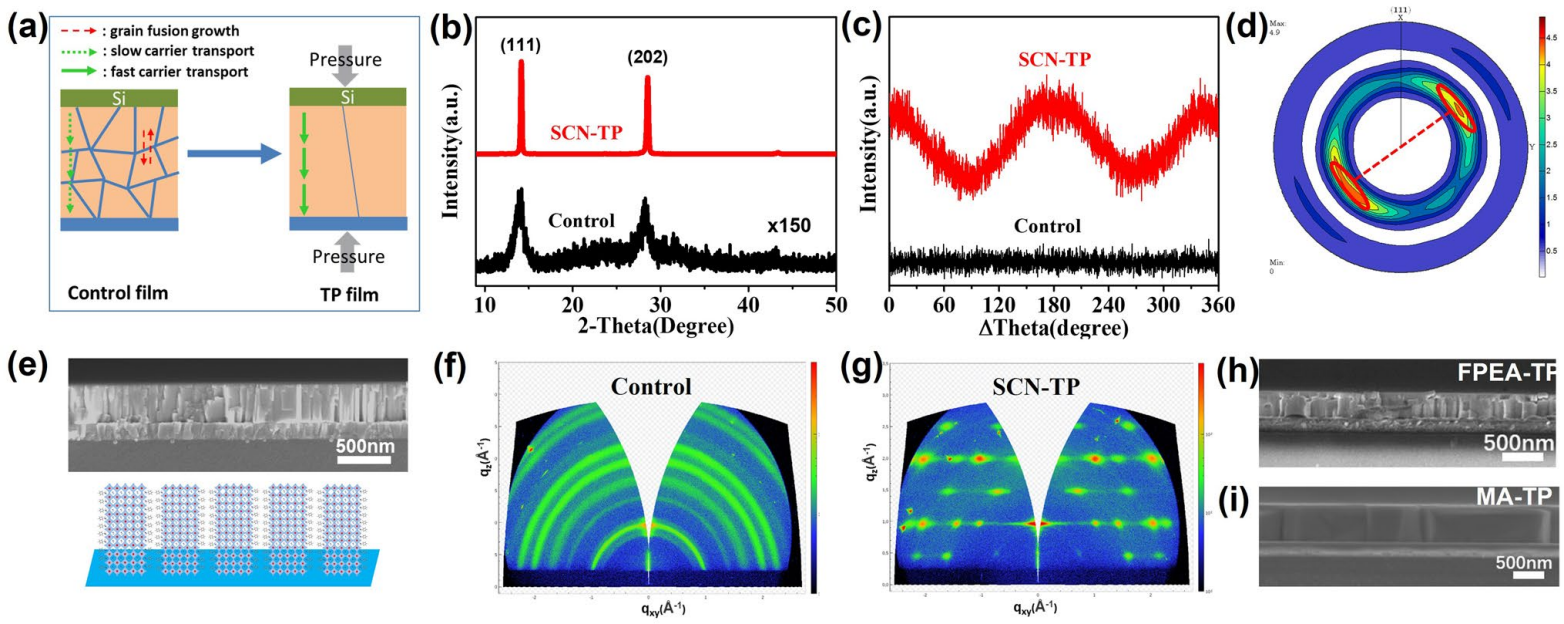
**a** Schematic diagram of thermal-pressure effect. **b** X-ray diffraction patterns and **c** Phi-scans of control and SCN-TP films. **d** Pole figure and **e** cross-sectional SEM image of SCN-TP film. GIWAXS images of **f** control film and **g** SCN-TP film. Cross-sectional SEM image of **h** FPEA-TP film and **i** MA-TP film

The positive effect of pressure for the crystal growth of perovskite grains is shown in Fig. 1a. Combining of the “soft” characteristic of perovskite grains, the uniform pressure along the vertical direction can drive the ion diffusion at the GBs (as red arrows labeled) to achieve fusion growth of adjacent grains. The formed single-crystal channel is favorable for fast carrier transport. First, taken PEA_2_MA_4_Pb_5_I_16_ thin film for example, optimizing uniaxial orientation was illustrated by the growth of thin film on ITO substrate directly, which could be designed as hole-free photodetector device. The control film is prepared by a conventional one-step spin-coating and annealing process. Because the SCN^−^ ions can be coordinated with Pb^2+^ ions in perovskite film, the crystallization and oriented growth of PEA_2_MA_4_Pb_5_I_16_ perovskite films can be regulated during annealing [27]. The NH_4_SCN additive can be removed completely after annealing (Fig. S1). Meanwhile, because high pressure could promote ion migration at solid-phase interface owing to the higher thermal energy [28], GBs fusion along the pressure direction could be expected to achieve single-grain penetration in the oriented thin films. The obtained PEA_2_MA_4_Pb_5_I_16_ thin film is named as SCN-TP film. The corresponding preparing process is schematically illustrated in Fig. S2.

X-ray diffraction (XRD) patterns are used to study the crystallinity and oriented feature of PEA_2_MA_4_Pb_5_I_16_ thin films. The main diffraction peaks at 14.1° and 28.4° correspond to (111) and (202) planes of 2DRP perovskite, respectively (Fig. 1b) [29]. The control film shows very low crystallinity as indicated by the weak and broad peaks. In contrast, the diffraction peaks of SCN-TP film show greatly enhanced intensity with a factor of ~ 150. Only two strong peaks can be observed in the XRD pattern of the SCN-TP film. These XRD result preliminarily demonstrates high crystallinity of the SCN-TP film [30]. Oriented growth measurements are further investigated. As shown in Fig. S3, the rocking curve around the growth axis of (111) plane of the control film displays no peak feature. In contrast, the SCN-TP film exhibits a sharp peak in the corresponding rocking curve, confirming favorable orientation ascribed to the positive effect of TP treatment. We further measure the orientation of the film through phi-scan measurement. The (202) plane is selected for test. The sample was rotated azimuthally from 0° to 360° (Fig. 1c). The phi-scan of the SCN-TP film shows a pair of strong peaks at the interval of ~ 180°, which corresponds to the twofold symmetry of (202) plane. On the contrary, no peak can be observed in the curve of the disorder control film. Furthermore, the orientation growth characteristic of the SCN-TP film is further demonstrated by the X-ray pole figure (Fig. 1d). Discrete two spots are separated azimuthally by 180°, which corresponds to a twofold symmetry axis [31].

Furthermore, cross-sectional scanning electron microscope (SEM) is performed to characterize the morphology of the film. For the SCN-TP film, the grains are grown into regularly columnar structure with uniaxially perpendicular orientation (Fig. 1e), which is much better than the control and SCN films (Fig. S4). It is conducive to charge transfer in the vertical direction, which is beneficial to enhanced performance of perovskite-based devices [32]. The orientation structure of the films was further measured by grazing incidence wide-angle X-ray scattering (GIWAXS). The 2D scattering pattern of the control film shows typical diffraction rings (Fig. 1f), indicating random orientation of the grains in the film. When NH_4_SCN additive was added in the precursor solution, the diffraction spots instead of rings appears, indicating the vertical orientation of the SCN film (Fig. S5). However, the spots are distorted and markedly elongated, which should be attributed to some degree of disordered characteristic. In contrast, for the SCN-TP film, the distortion and elongation of the diffraction spots are greatly mitigated (Fig. 1g), which demonstrates enhanced orientation structure [33]. Meanwhile, owing to pressure-enhanced ion diffusion, fusion growth of the grains and isostatic pressure on the SCN-TP film, the pinhole-free surface with ultralow root mean square roughness (RMS: 0.49 nm) is also achieved (Fig. S6). The smooth surface is also favorable for the formation of uniform spin-coated transport layer, thereby improving the interface contact between active layer and transport layer, and then benefiting for the carrier transport.

For demonstrating the relative universality of the TP-enhanced orientation growth strategy, other 2D and 3D perovskite systems were also investigated. Interestingly, analogous results are emerged. Then, the typical quasi-2D FPEA_2_MA_4_Pb_5_I_16_ and 3D MAPbI_3_ thin films grown on the PEDOT: PSS layers via the TP growth strategy are named as FPEA-TP (Fig. 1h) and MA-TP (Fig. 1i) films, respectively. Taken MAPbI_3_ for example, the effect of pressure on the crystal growth of perovskite grains is simply illustrated. As the side-view images shown in Figs. 1i and S7, when pressure along the vertical direction is applied on the MA-control film, the disordered multicrystal structure along thickness direction (Fig. S7) converts into single-grain penetration, forming regularly columnar structure (Fig. 1i). In addition, as the top-view images shown in Fig. S8, the MA-control film is composed of small grains. After pressure-induced fusion growth, the lateral grain size of MA-TP film is markedly increased (Fig. S8). The GB and trap-state concentration are effectively reduced, which is potentially conducive to improving the carrier transport efficiency and the stability of thin film devices. The uniaxial orientation of them is also demonstrated via the corresponding XRD patterns and rocking curves (Fig. S9).

**Fig. 2:**
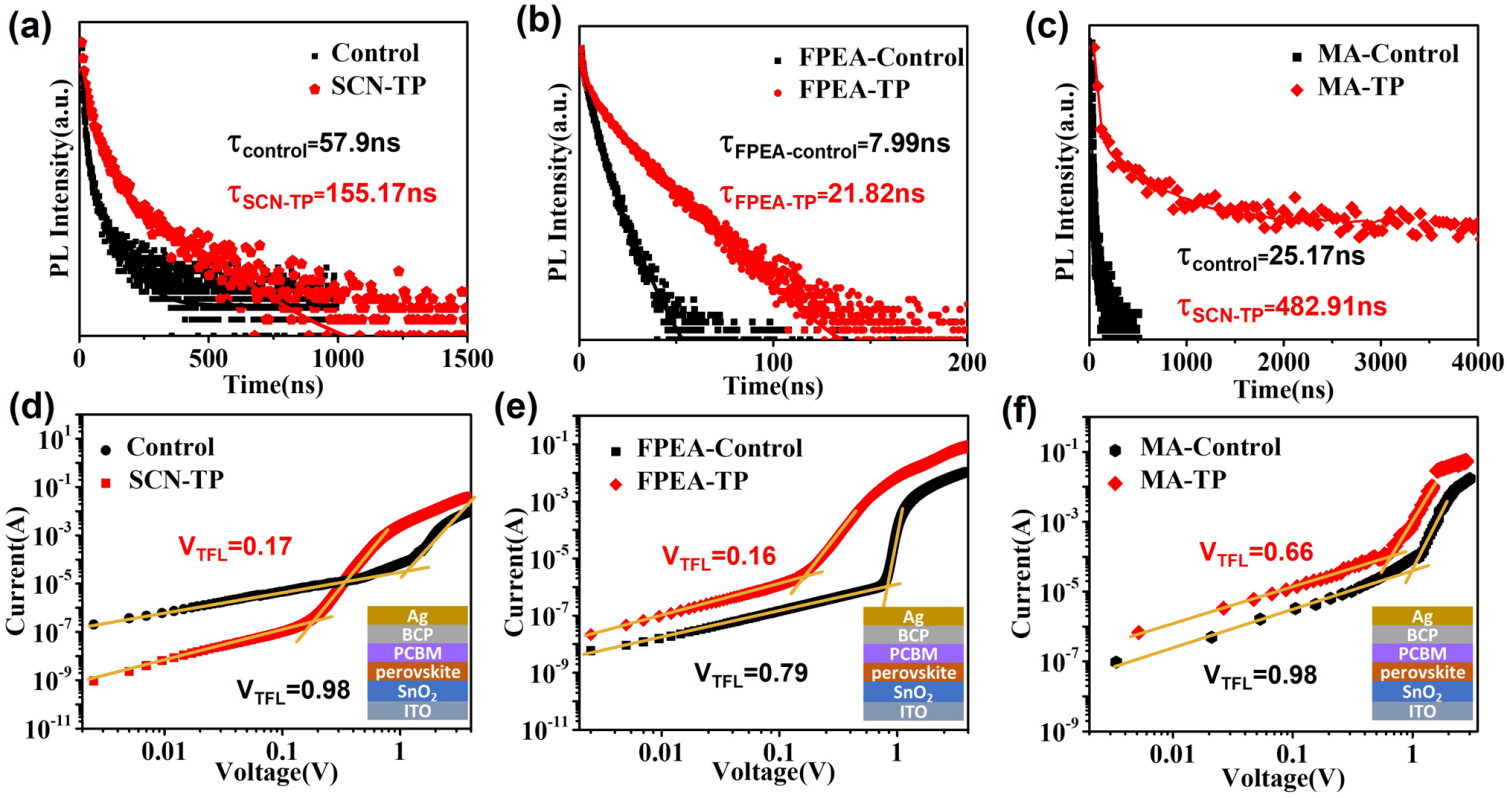
Time-resolved PL decay curves of **a** PEA-based films** b** FPEA-based films and **c** MA-based films. The electron-only devices of **d** PEA-based films** e** FPEA-based films and **f** MA-based films

The photo-physical properties of these films are further investigated. The carrier recombination dynamics of these films are studied by time-resolved photoluminescence (TRPL). The fast and slow decays are related to the quenching of charge transfer at the interface and the recombination of free carriers, respectively. As shown in Fig. 2a–c, the average recombination lifetimes (*τ*_avg_) of the control films are 57.9 ns (PEA-control), 7.99 ns (FPEA-control) and 25.17 ns (MA-control), respectively. In contrast, the *τ*_*avg*_ values of the corresponding TP films are markedly improved to 155.17, 21.82 and 482.91 ns, respectively, which agrees with the enhanced uniaxial orientation and crystallinity [34]. The significantly increased *τ*_avg_ demonstrates the remarkable reduction of trap-state density, determining excellent photoelectrical performance of the TP film devices. Furthermore, the trap-state densities (*N*_trap_) of these films are evaluated by space charge limited current (*SCLC*) analysis with the following equation [35]:

1 $$N_{{{\text{trap}}}} = 2\varepsilon_{0} \varepsilon_{r} V_{{{\text{TFL}}}} /qL^{2}$$

where *V*_TFL_ is the onset voltage of the trap-filled limit region, *ε*_0_, *ε*_*r*_ are the vacuum dielectric constant and the relative dielectric constant, *q* is the elementary charge, *L* is the thickness of the film. Electron-only devices (ITO/SnO_2_/perovskite/PCBM/BCP/Ag) are designed. Figure 2d–f shows the corresponding dark *J-V* characteristics of them. Clearly, when the low bias voltage exceeds trap-filled limit, a sharp increase is followed for the *J-V* curves. According to the equation, the calculated *N*_trap_ values of the control films are 3.17 × 10^16^ cm^−3^ (PEA-control), 7.5 × 10^15^ cm^−3^ (FPEA-control) and 6.85 × 10^15^ cm^−3^ (MA-control), respectively. In comparison, the calculated *N*_*trap*_ values of the corresponding TP films decreases sharply to 4.87 × 10^15^, 3.45 × 10^15^, and 4.6 × 10^15^ cm^−3^, respectively, which should be ascribed to the enhancement of crystallinity, the removal of GBs especially along the vertical direction and reduced surface defects due to greatly lowered surface roughness. Thus, suppressed non-radiative recombination loss should be expected for excellent photoresponse in the device.

### Characterization of Perovskite Film Devices

**Fig. 3:**
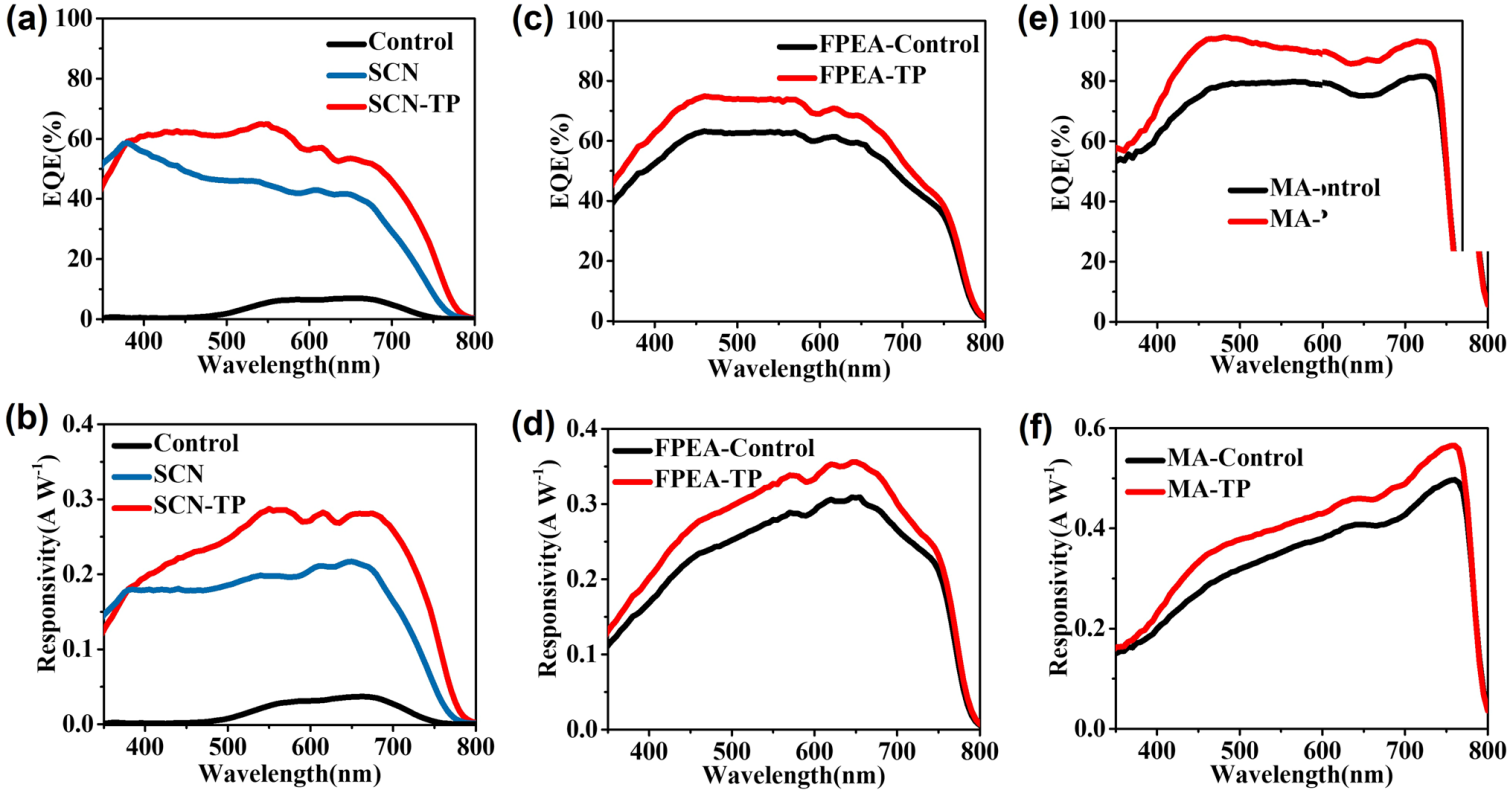
**a** EQE of PEA-based devices. **b** *R*_*λ*_ of PEA-based devices. **c** EQE of FPEA-based devices and **d** *R*_*λ*_ of FPEA-based devices. **e** EQE of MA-based devices. **f** *R*_*λ*_ of MA-based devices

To illustrate the positive effect of ordered carrier transport for the photoresponse enhancement of perovskite film-based devices, the device configurations were adopted (Fig. S10). Because the SCN-TP film was directly grown on ITO substrate, a hole-free 2DRP perovskite device was designed. The optical transmittance spectrum of ITO substrate (Fig. S11) shows uniform and high transmittance in visible region, demonstrating that the influence of ITO substrate for light absorption of perovskite films can be ignored. The external quantum efficiency (*EQE*) and spectral responsivity (*R*_*λ*_) are two important factors to assess the photodetector performance. The *EQE* and *R*_*λ*_ can be calculated by the following equations [36]:

2 $${\text{EQE}} = \left( {\Delta I/q} \right)\left( {{\text{PA}}/{\text{h}}v} \right)$$

3 $$R_{\lambda } = {\text{EQE}}\left( {q/{\text{h}}v} \right)$$

where Δ*I* is the photocurrent, *q* is elementary charge, *P* is the illumination light intensity, *A* the effective illuminated area, h is the Planck constant and *ν* is the frequency of incident light. Figure 3a shows that the self-powered device based on the SCN-TP thin film exhibits dominantly improved EQE compared to those of the control and SCN films. Although the 2DRP perovskite device is hole-free, the self-powered SCN-TP film photodetectors still present a broad photoresponse with EQE over 50% from 370 to 700 nm, which is mainly ascribed to high-quality perovskite film. Based on the *EQE* spectrum of the SCN-TP film device, the calculated *R*_*λ*_ of the self-powered photodetector at different wavelength is shown in Fig. 3b. It exhibits a *R*_*λ*_ over 0.2 A W^−1^ at a broad range of 400–750 nm with a peak value of 0.29 A W^−1^ at 550 nm, which is much better than the peak values of the control (0.03 A W^−1^) and SCN (0.2 A W^−1^) devices. This hole-free SCN-TP photodetector is comparable to and even better than the reported perovskite works (Table S1). These results preliminarily demonstrate the positive effect of optimized film structure for self-powered photoresponse behavior.

However, compared to PEA in 2DRP perovskite films, FPEA has a larger steric hindrance contribution [37], allowing it to pack more ordered cation layers and form a more extended structure than PEA. FPEA can enhance the orbital interaction between the inorganic layers compared to PEA, resulting in more effective charge transfer through adjacent inorganic layers [38]. The lack of electronous F atoms and the interlayer electrostatic attraction between the adjacent benzene rings is arranged along with the confirmed dipole interaction [39]. Thus, combining of the TP oriented growth strategy, more excellent photoresponse performance can be expected for the 2DRP perovskite-based SPPDs after PEA was replaced by FPEA. In addition, the PEDOT: PSS is one of the most important HTL materials in inverted devices because of its high conductivity, good transparency in the visible range, good coverage, adjustable wettability, etc. [40]. The planar heterojunction device of ITO/PEDOT: PSS/perovskite/PCBM/BCP/Ag was adopted as illustrated in **Fig. S12**. The *EQE* spectrum and corresponding *R*_*λ*_ at different wavelength of the FPEA-TP device are shown in Fig. 3c, d. Based on the *EQE* spectrum, the calculated maximum *R*_*λ*_ is 0.36 A W^−1^ at 650 nm, which is higher than that of the FPEA-control device. The *R*_*λ*_ value greater than 0.31 A W^−1^ is also observed in a wide wavelength range from 500 to 700 nm. Furthermore, the device configuration of ITO/PEDOT: PSS/MAPbI_3_/PC_61_BM/Bphen/Ag structure was also adopted to study its self-powered photoresponse performance. As shown in Fig. 3e, f, compared to the MA-control film, the *EQE* of the MA-TP film SPPD is significantly improved. More significantly, the *EQE* value exceeds 85% in a broad range from 430 to 770 nm with a maximum value of 93.2% at 760 nm that is much higher than that of the MA-control film (maximum: 81.7%). The wide absorption range is conducive to the generation and separation of a large number of electrons and holes. Based on the *EQE* spectrum, the calculated maximum *R*_*λ*_ of MA-TP based SPPDs is 0.57 A W^−1^ at 760 nm, which is higher than that of the MA-control device and even much close to the theoretical value (0.61 A W^−1^), demonstrating giant photoelectrical performance. As reported, the self-powered behavior of the MA-TP film has been one of the best results among the perovskite-based SPPDs (Table S2). For these TP-based SPPDs, the excellent photoresponse behaviors should be attributed to longer charge carrier lifetime and lower trap density (Fig. 2). In addition, the Nyquist plots were also investigated to demonstrate the superior photoresponse behaviors (Fig. S13). Charge carrier transport of the MA-TP photodetectors is also enhanced and the charge recombination process is effectively reduced. Thus, for the 2D and 3D perovskite films, the TP effect is universally favorable for charge carrier transport and suppresses nonradiative recombination loss to achieve more excellent photoresponse.

**Fig. 4:**
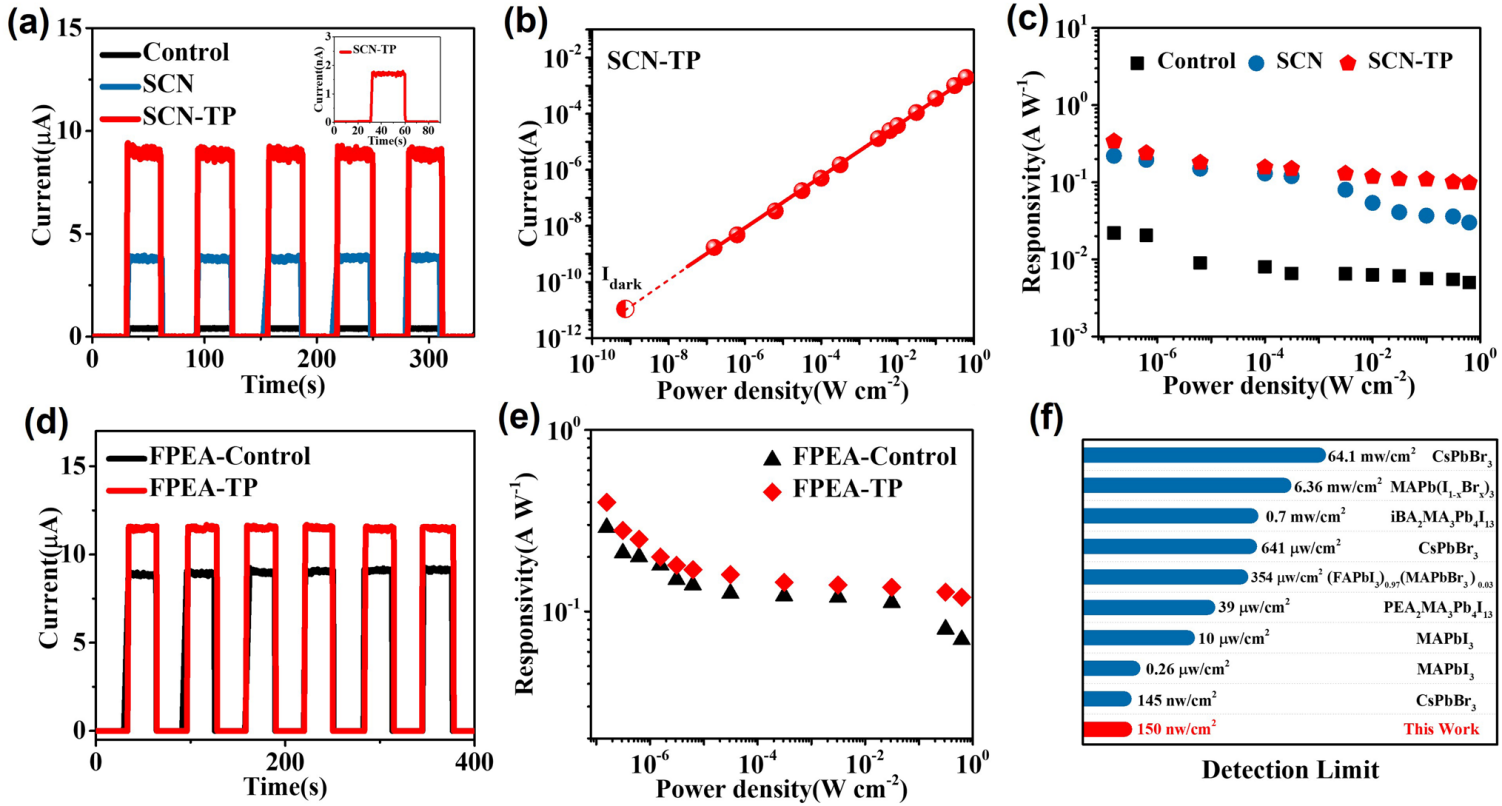
**a** Transient photoresponse properties of PEA-based photodetectors for 532 nm of 1 mW cm^−2^ with 0 bias. **b** Linear dynamic range of SCN-TP photodetector. **c** *R* values of PEA-based photodetectors. **d** Transient photoresponse properties of FPEA-based photodetectors for 532 nm of 1 mw cm^−2^ with 0 bias. **e** *R* values of FPEA-based photodetectors. **f** Detection limit of different photodetectors

Furthermore, the self-powered photoresponse behaviors based on the SCN-TP and FPEA-TP perovskite thin films were further studied under 532 nm laser illumination. As shown in Fig. 4a, the SCN-TP device exhibits a significantly improved photocurrent. At a power density of 625 mW cm^−2^, the photocurrent is as high as 10^–3^ A (Fig. 4b). Accordingly, the SCN-TP SPPD displays an ultrahigh on/off ratio (1.8 × 10^8^) at 625 mW cm^−2^ and 0 V (Fig. S14), which is more superior than these of the control and SCN devices. In the practical application of photodetectors, the photocurrent is required to be proportional to the power density and has a wide response range. It is usually characterized by linear dynamic range (*LDR*), which is given by the following formula [41, 42]:

4 $${\text{LDR}} = 20{\text{log}}\frac{{I_{{{\text{ph}}}} }}{{I_{{\text{d}}} }}$$

where *I*_ph_ is the photocurrent and *I*_d_ is the dark current. The measured LDR value of the SCN-TP device calculated by the dark current (11 pA) is as high as 165.1 dB (Fig. 4b). Furthermore, the responsivity (*R*) is calculated via the following equation [43, 44]:

5 $${\text{R}} = \left( {I_{ph} - I_{d} } \right)/{\text{PA}}$$

where *B* is modulation frequency, and* i*_*n*_ is noise current dominated by *1/f* noise (Fig. S15). When the power density is as low as 150 nW cm^−2^, the *R* value is as high as 0.34 A W^˗1^ (Fig. 4c), which are more superior than those of the control (0.022 A W^˗1^) and SCN (0.22 A W^˗1^) devices. Besides, the SCN-TP device also exhibits rapid response rate (Fig. S16). In addition, the FPEA-TP device also exhibits a markedly improved photocurrent, which is even higher than that of the PEA-TP SPPD (Figs. 4d and S17). The measured *LDR* value calculated by the dark current (0.17 nA) is as high as 143 dB (Fig. S18), exhibiting a wide linear photoresponse range from 150 nW cm^−2^ to 625 mW cm^−2^. In Fig. 4e, R value is also calculated for further evaluating the device performance with a modulation frequency of 100 Hz (Fig. S19). When the power density reduces to 150 nW cm^−2^, the corresponding *R* value is as high as 0.4 A W^˗1^, which is more excellent than mostly reported perovskite-based SPPDs (Table S3). The *R* value of FPEA-control is only 0.29 A W^−1^. Meanwhile, the performance of the FPEA-TP devices exhibit excellent repeatability (Fig. S20). Although the NH_4_SCN and DMSO additives have been demonstrated to effectively prompt oriented growth of 2DRP perovskite films, when TP growth strategy was further conducted on the FPEA-control film, persistently enhanced responsivity was achieved for the FPEA-TP film device. The FPEA-TP device also presents rapid response rate due to ordered charge carriers transport and interface extraction abilities (Fig. S21). The detection limit is also a critical parameter of SPPDs for practical applications, which refers to the acquisition ability of weak optical signal [45]. Interestingly, the detection limit of 150 nW cm^−2^ is lower than mostly reported perovskite-based SPPDs (Fig. 4f). The inset in Fig. 4a shows that although the power density is as low as 150 nW cm^−2^, obvious current enhancement is still exhibited between the corresponding photocurrent (1.7 nA) and dark current. Thus, a detection limit lower than 150 nW cm^−2^ can even be expected, which can be potentially applied in the weak optical detection.

The excellent performance of TP-based devices derived from ordered carrier transport should be closely related with the film structure and quality. Firstly, the TP films exhibit single-crystal-like smooth surface and single-grain penetration in the film thickness direction, which can limit the leakage current and cause low dark current [46]. It can improve the photoconductivity and transportation for photogenerated carriers. Secondly, because the smooth surface and increased crystallinity facilitate the reduction of the surface traps and the limitation of nonradiative recombination, the leakage of unfavorable electrons can be minimized [46]. Thirdly, the low trap-state density (Fig. 2) is also favorable for high photocurrent as well as low noise current (Figs. S16 and S19). Fourthly, compared to the control film, the TP film can effectively improve the carrier lifetime and transport (Fig. 2), minimizing the carrier recombination in the active layers. Thus, excellent photoelectrical performance is achieved in the TP-based self-powered photodetectors.

**Fig. 5:**
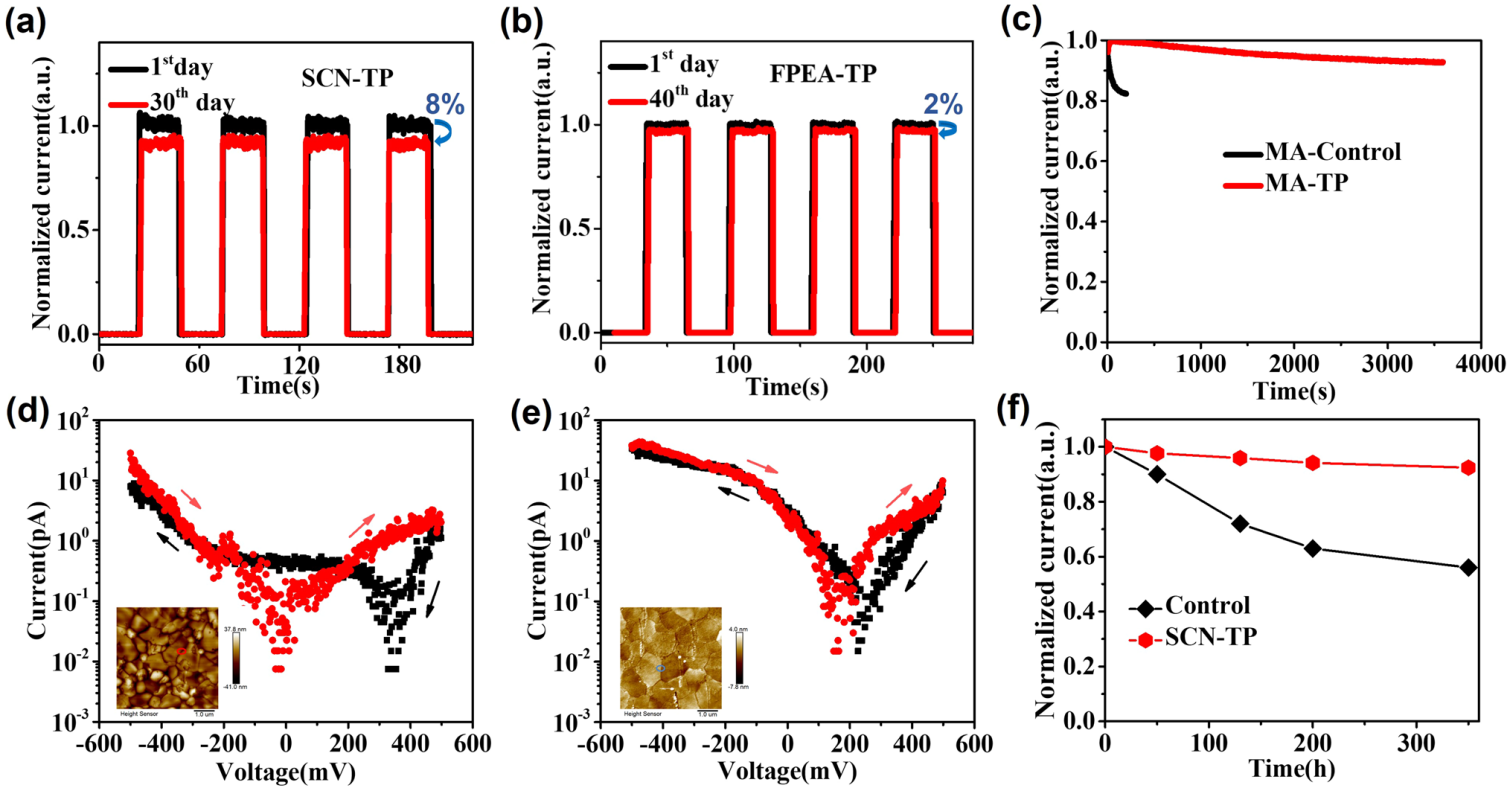
**a** SCN-TP and **b** FPEA-TP photodetectors without encapsulation in ambient air. **c** Light stability of MA-based photodetectors. *I-V* curves at the grain boundary of **d** MA-control film and **e** MA-TP film. **f** Thermal stability of PEA-based photodetectors at 60 °C

The effective removal and fusion of GBs in the perovskite films with single-crystal-like surface can be reasonably expected to improve the films and corresponding devices stability. Thus, representative TP films were selected to investigate the humidity, optical and thermal stabilities, respectively. First, SCN-TP device without any encapsulation and protection indeed exhibits stable photocurrent in an environment with a relative humidity of 30% within 30 days (Fig. 5a). The photocurrent attenuation is only 8% after 30 days. On the contrary, the photocurrent of the control film device can be significantly reduced by 50% 30 days later (Fig. S22). Correspondingly, the XRD patterns of the SCN-TP and control films maintained for 1 and 30 days, respectively, are investigated (Fig. S23). There is an obvious peak at 12.6° for the control film after 30 days, confirming part degradation of perovskite into PbI_2_. In contrast, the SCN-TP film kept excellent humidity stability after 30 days and no new impurity phase appeared. The similar phase and photoresponse stabilities were also observed in the FPEA-TP film and device in an environment with a relative humidity of 40% within 40 days (Figs. 5b and S24). The efficient improvement for humidity resistance should be attributed to the improved hydrophobicity of the TP film (Fig. S25), which could be promising to retard the degradation of device performance. In addition, during the on/off cycles, the hardly fluctuated photocurrent of the FPEA-TP device also demonstrates excellent operational durability of it (Fig. S26). Second, take MA-TP film for example, although the TP device is continuously illuminated, it still exhibits relatively stable photoresponse. As shown in Fig. 5c, the current of MA-control device degrades significantly for 18% of the initial value after 200 s continuous illumination. In contrast, that of the MA-TP device maintains superior stability after 200 s and only decreased ~ 6% even after continuous illumination for 3,600 s. Ion migration under illumination is one of the reasons of optical instability of perovskite films. As reported [47], ion migration in polycrystalline perovskite films dominates through GBs, inducing strong hysteresis both for photocurrent and dark current at the GBs. Thus, we measured the local current hysteresis in grain and at GBs using a conductive atomic force microscope (c-AFM). A large current hysteresis would commonly demonstrate a fast ion migration locally during the current scanning process. Figure S27 shows the c-AFM setup for current measurement. The dark current measured at the GBs area of the MA-control film shows clear hysteresis (Fig. 5d). In sharp contrast, the measured GB dark current in the MA-TP film shows negligible hysteresis (Fig. 5e), which is commonly observed in grain interior and further verified by that in grain of the MA-control film (Fig. S28). Thus, the ion migration at GBs area is effectively suppressed by the TP strategy, which drives secondary grain growth and deep fusion of the adjacent GBs, thereby enhancing the optical stability of the TP-based perovskite device. Third, thermal stability of perovskite film device was also investigated. In the testing procedure, the non-encapsulated perovskite-based SPPDs were heated continuously at 60 °C for a different time. After that, these preheated devices were immediately tested in an environment with 20 °C and a relative humidity of 30%. When the SCN-TP film photodetectors were heated for 350 h under 60 °C, the photocurrent attenuation was only ~ 8% (Fig. 5f). On the contrary, that of the control film device was significantly reduced to 56% of the initial photocurrent. It should be attributed to the crystallinity improvement of the TP film markedly. Visibly, these TP films and devices all exhibit superior environmental stability.

## Conclusions

In summary, uniaxial orientation of quasi-2D and 3D perovskite thin films directly grown on ITO glass and transport layers, respectively, were all realized through the general TP growth strategy. Pressure-enhanced ion diffusion effect promotes grain boundaries fusion along the pressure direction. The formed single-grain penetration in the thickness direction is crucial for the ordered carrier transport and electron–hole separation in photovoltaic self-powered device. Combining of the improved crystallinity, self-fused boundaries, longer carrier lifetime and lower trap-state density, the optimized TP-based self-powered photodetectors exhibit high responsivity in a wide wavelength range under white light and laser illumination, respectively. Moreover, they all present overall improved stability, reproducibility, ultralow detection limit and fast response. Thus, this work provides a universal opportunity to achieve uniaxial orientation of perovskite thin films to construct efficient and stable self-powered photodetectors and other photoelectrical devices.

### Supplementary Information

Below is the link to the electronic supplementary material.

Supplementary file1 (PDF 1472 KB)
