# Supplementary material for: A General Strategy for Ordered Carrier Transport of Quasi-2D and 3D Perovskite Films for Giant Self-Powered Photoresponse and Ultrahigh Stability
Source: Nanomicro Lett. 2023 Apr 30;15:115. doi: 10.1007/s40820-023-01087-5 (PMC10149430; doi:10.1007/s40820-023-01087-5)
Supplement: Supplementary file 1 — Supplementary file1 (PDF 1472 KB) [file 40820_2023_1087_MOESM1_ESM.pdf]

Supporting Information for

A General Strategy for Ordered Carrier Transport of Quasi-2D and 3D Perovskite Films for Giant Self-Powered Photoresponse and Ultrahigh Stability

Fei Zhu[1], Gang Lian[1, *], Deliang Cui[1], Qilong Wang[2], Haohai Yu[1, *], Huaijin Zhang[1], Qingbo Meng[3], Ching-Ping Wong[4, *]

[1]State Key Laboratory of Crystal Materials, Shandong University, Jinan 250100, P. R. China

[2]Key Laboratory for Special Functional Aggregated Materials of Education Ministry, School of Chemistry & Chemical Engineering, Shandong University, Jinan 250100, P. R. China

[3]Key Laboratory for Renewable Energy, Chinese Academy of Sciences (CAS), Beijing Key Laboratory for New Energy Materials and Devices, Beijing National Laboratory for Condensed Matter Physics, Institute of Physics, CAS, Beijing 100190, P. R. China

[4]School of Materials Science and Engineering, Georgia Institute of Technology, Atlanta, Georgia, 30332, United States

*Corresponding authors. E-mail: liangang@sdu.edu.cn (Gang Lian), haohaiyu@sdu.edu.cn (Haohai Yu), cp.wong@mse.gatech.edu (Ching-Ping Wong)

Supplementary Figures and Tables

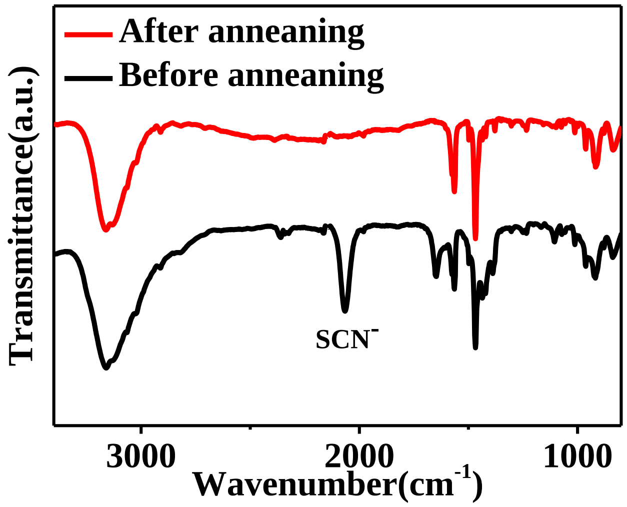

Fig. S1 FTIR spectra of the perovskite SCN films before and after annealing

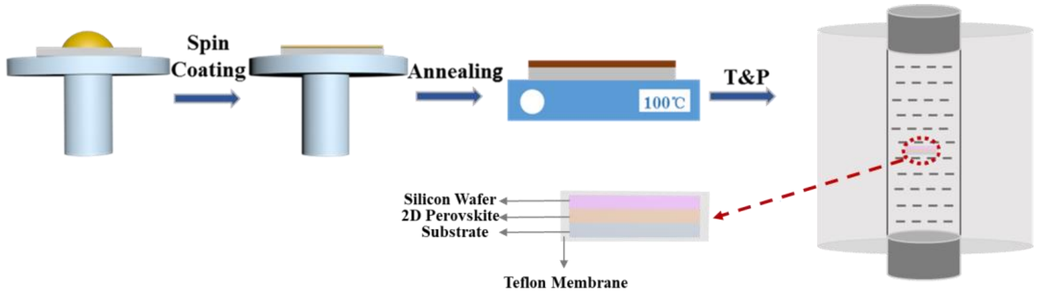

Fig. S2 Schematic illustration of perovskite film by TP method

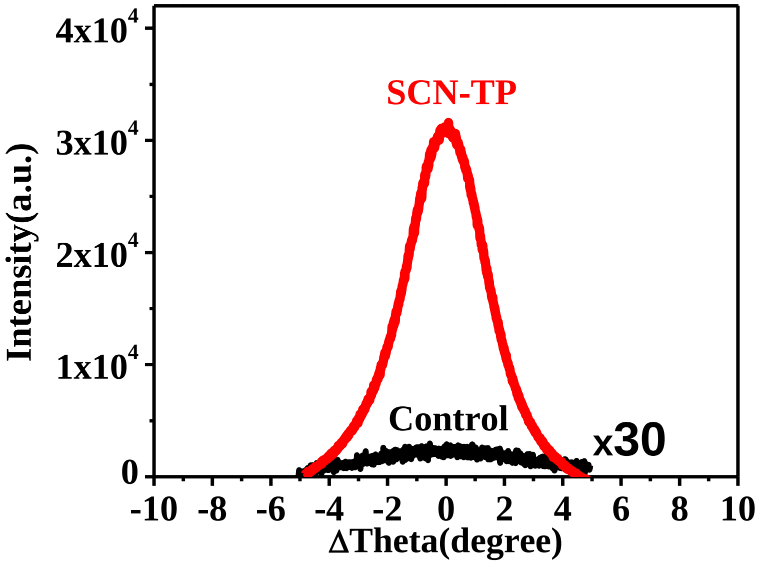

Fig. S3 Rocking curves of control and SCN-TP films

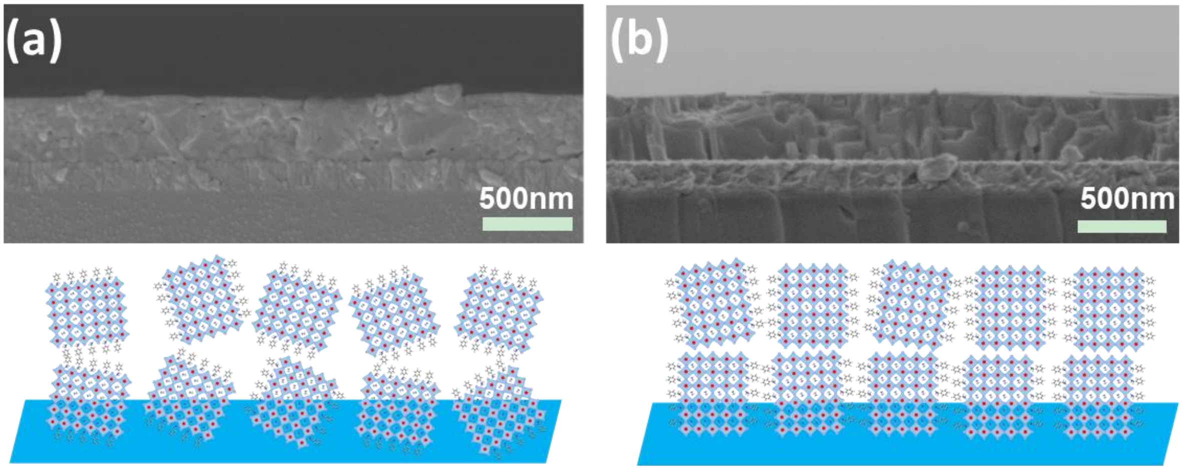

Fig. S4 Cross-sectional SEM images of perovskite **a** control film and **b** SCN film

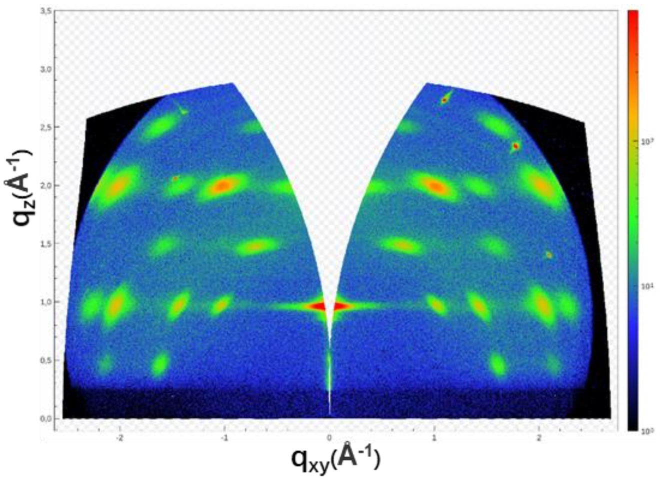

Fig. S5 GIWAXS image of the SCN film

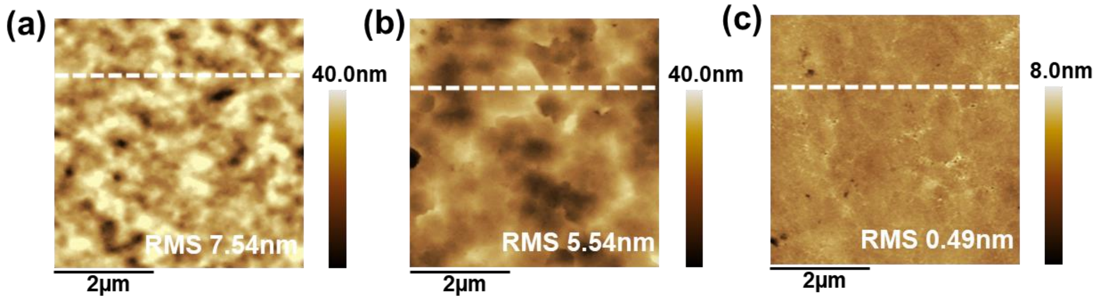

Fig. S6 AFM topography images of perovskite films: **a** control **b** SCN **c** SCN-TP films

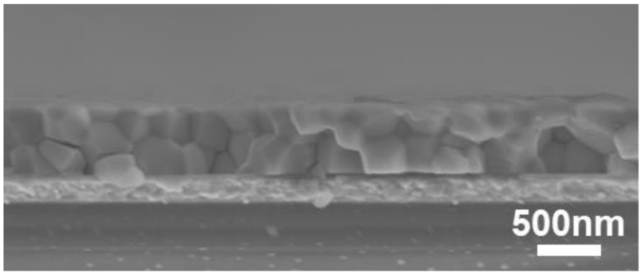

Fig. S7 Side-view SEM image of the MA-control film

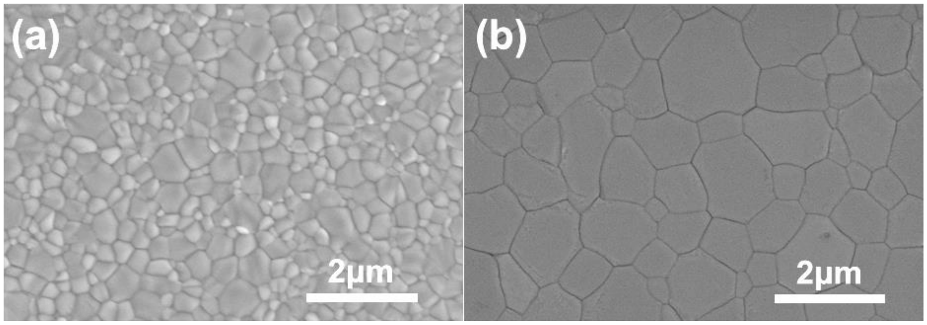

Fig. S8 Top-view SEM images of **a** MA-control and **b** MA-TP films

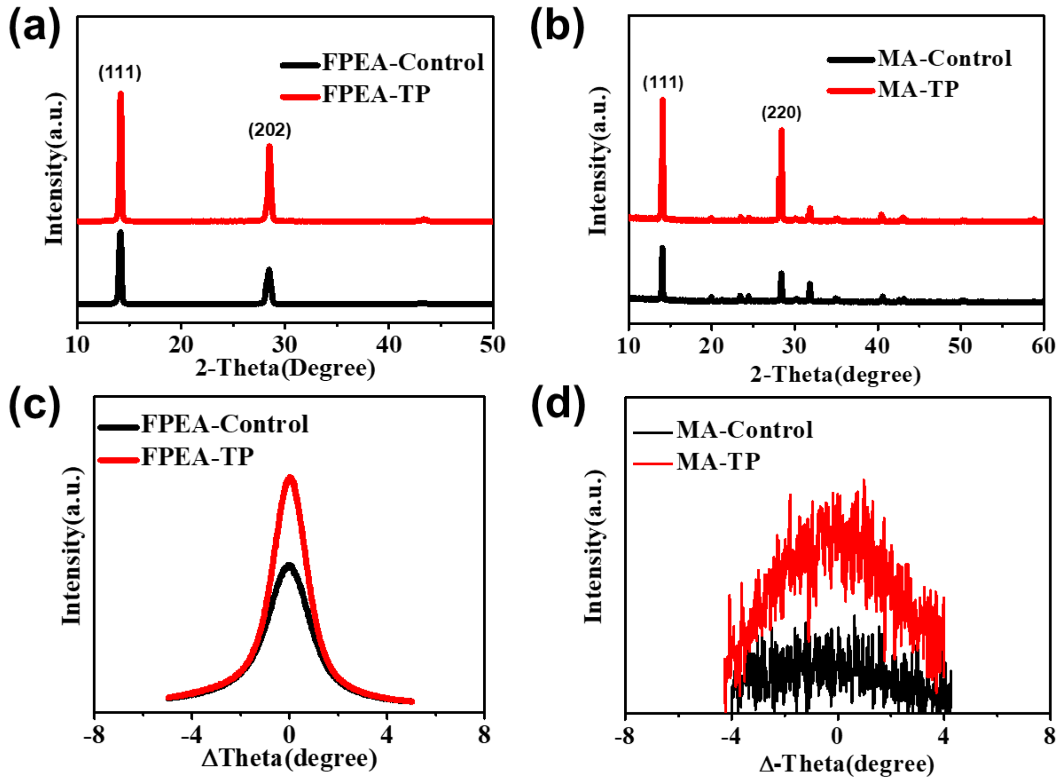

Fig. S9 XRD patterns of **a** FPEA-based films and **b** MA-based films. Rocking curves of **c** FPEA-based films and **d** MA-based films

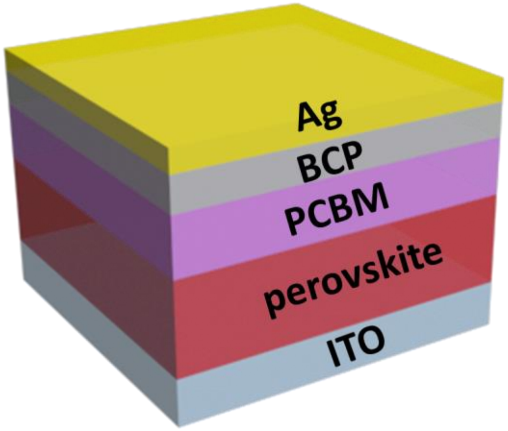

Fig. S10 Device structure of hole-free $(PEA)_2(MA)_4Pb_5I_{16}$ film

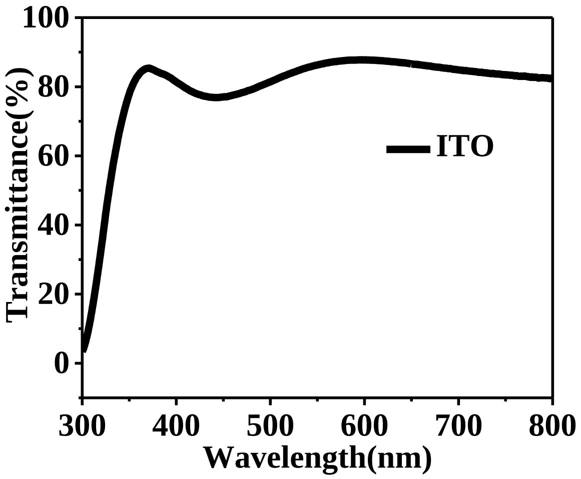

Fig. S11 Optical transmittance spectrum of the ITO substrate

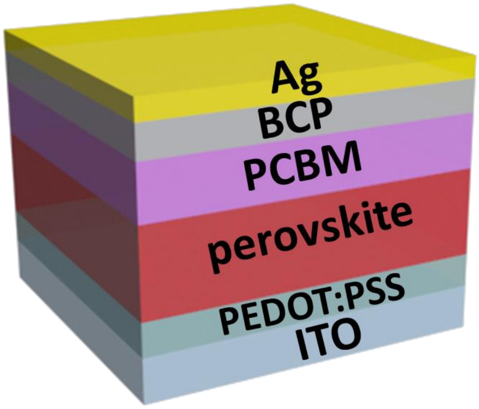

Fig. S12 Device structure of $(FPEA)_2(MA)_4Pb_5I_{16}$ film

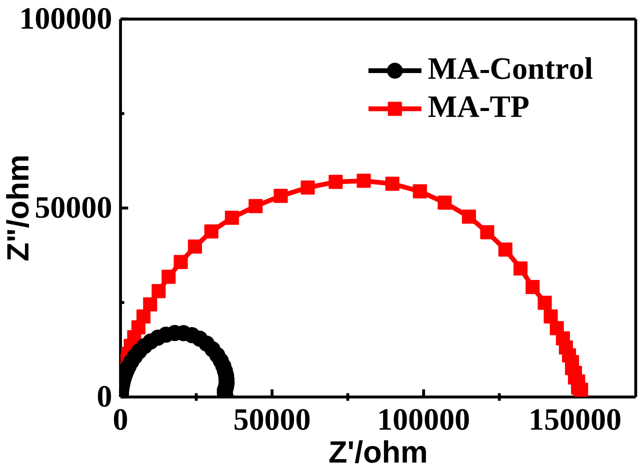

Fig. S13 Nyquist plots of MA-based photodetectors in the dark

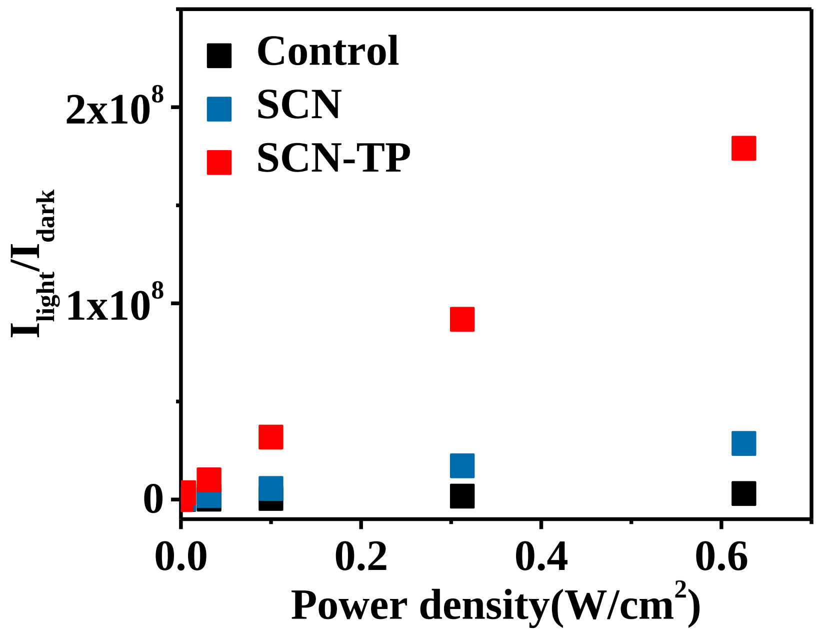

Fig. S14 I_{light}/I_{dark} ratio of the photodetectors

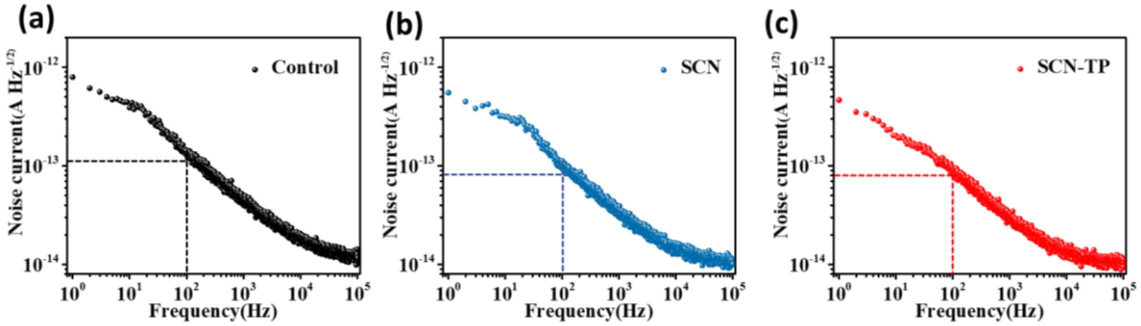

Fig. S15 Frequency-dependent noise current at a bias voltage of 0 V

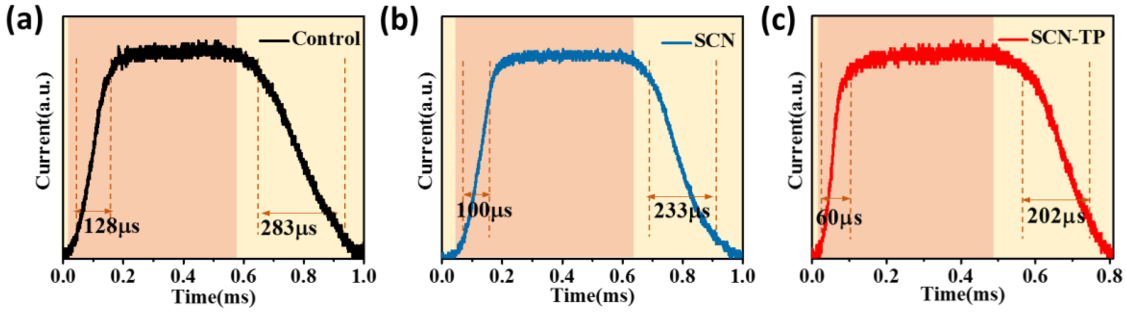

Fig. S16 Current-time curves of **a** control **b** SCN and **c** SCN-TP photodetectors

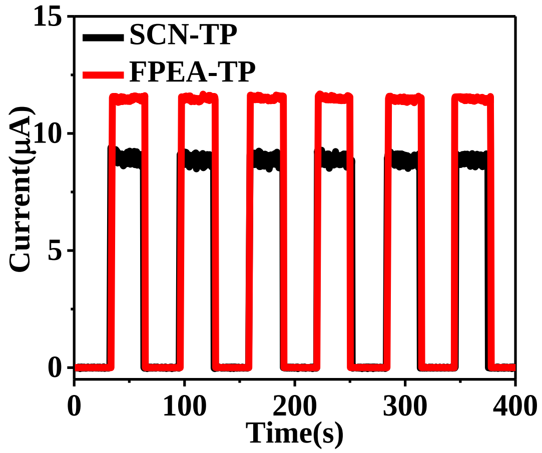

Fig. S17 Transient photoresponse properties based on SCN-TP and FPEA-TP films for 532nm of 1mW cm^{-2} with 0 bias

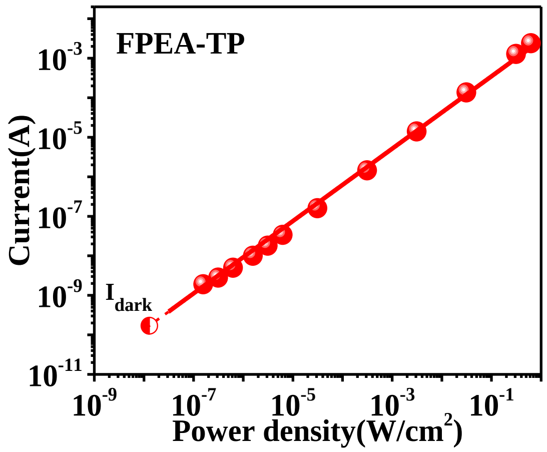

Fig. S18 Linear dynamic range of FPEA-TP photodetector

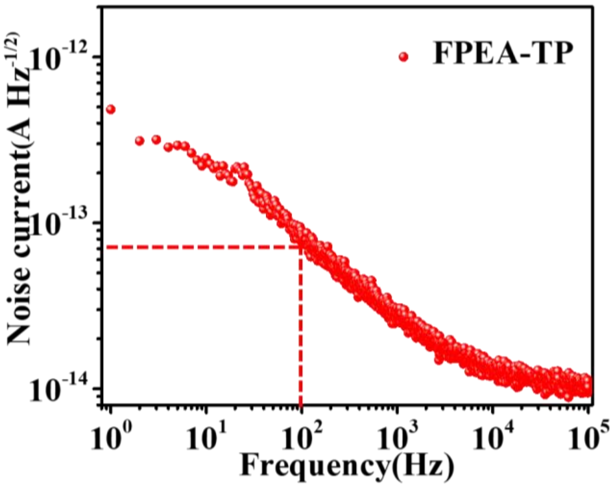

Fig. S19 Frequency-dependent noise current at a bias voltage of 0 V of FPEA-TP photodetector

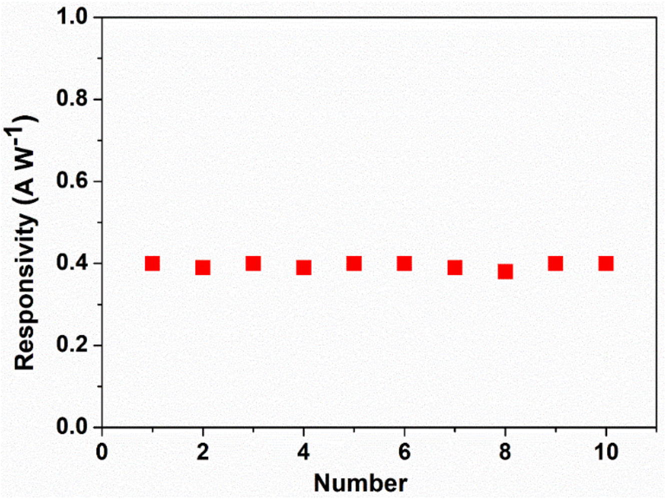

Fig. S20 Repeatability measurement of FPEA-TP photodetector

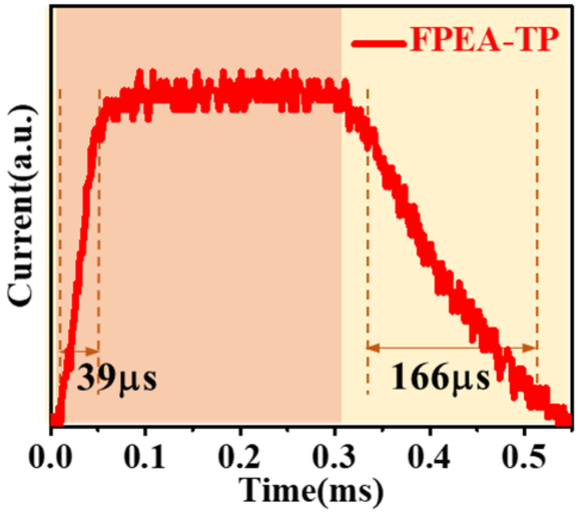

Fig. S21 Current-time curve of the FPEA-TP photodetector

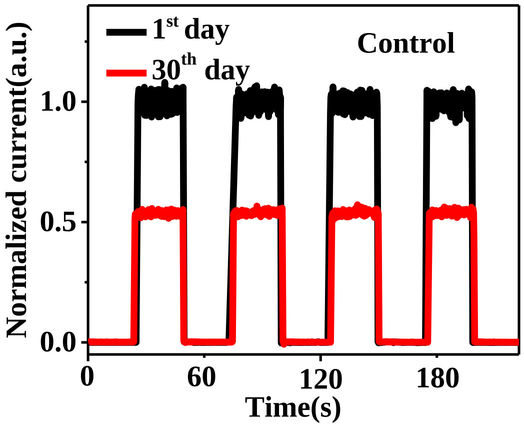

Fig. S22 Normalized photocurrents of control photodetectors without encapsulation in ambient air with relative humidity of 30%

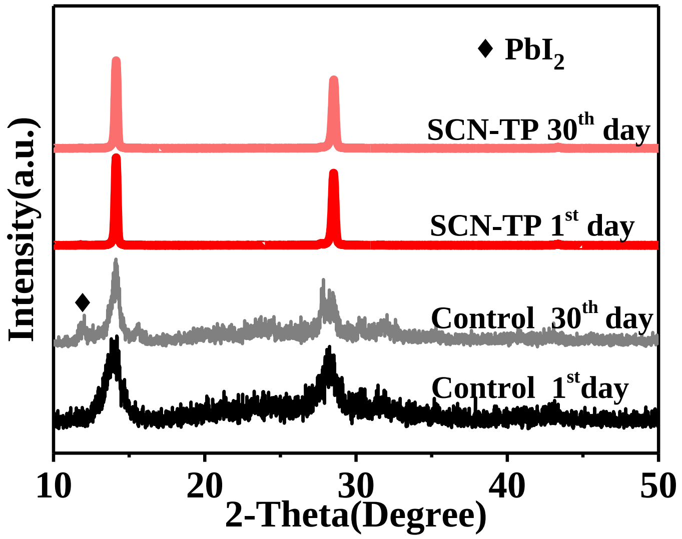

Fig. S23 XRD patterns of the SCN-TP and control photodetectors without encapsulation in ambient air with relative humidity of 30%

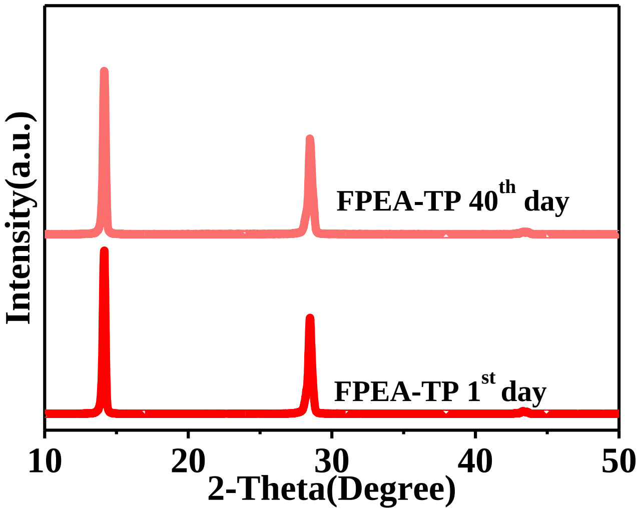

Fig. S24 XRD patterns of the FPEA-TP photodetectors without encapsulation in ambient air with relative humidity of 40%

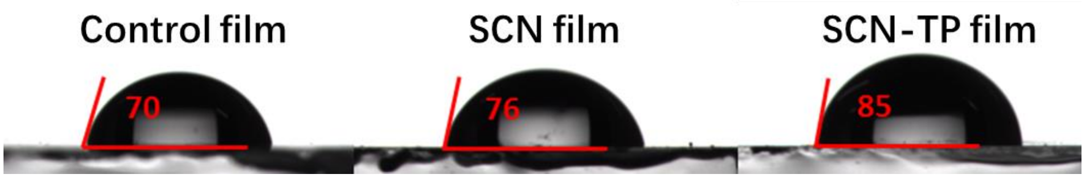

Fig. S25 Contact angles of the control, SCN and SCN-TP films

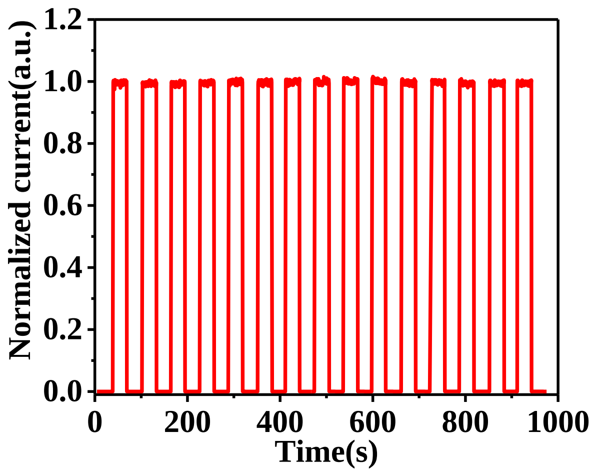

Fig. S26 Operational durability measurement of the FPEA-TP photodetector

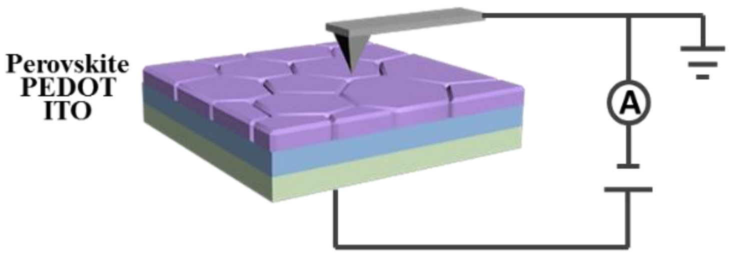

Fig. S27 c-AFM setup for current measurement.

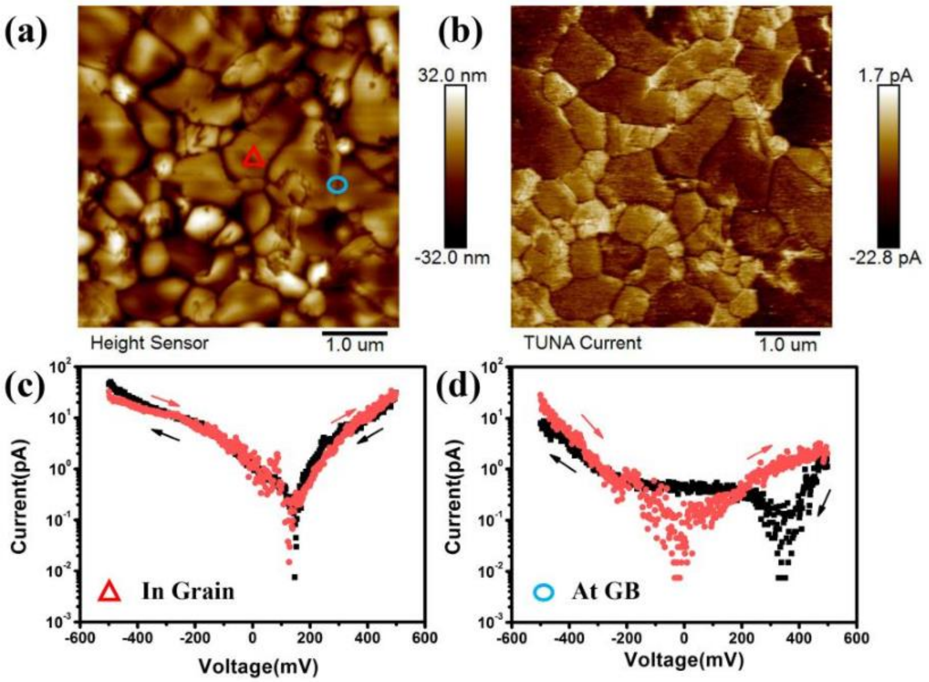

Fig. S28 AFM image and I-V curves in grain and at grain boundary of the MA-control film

Table S1 Summary of R_λ based on self-powered hole-free device

Photodetector Structure	Responsivity (A W^{-1})	Wavelength (nm)	Refs.
ITO/$CsPbBr_3$/Au	0.2	500	S1
ITO/SnO_2/$CsBi_3I_{10}$/Au	0.2	650	S2
Au/Bi-$MAPbCl_3$/$MAPbBr_3$/Au	0.038	500	S3
ITO/$CH_3NH_3PbCl_3$/ PTAA/Al	0.047	398	S4
FTO/ $CsBi_2I_9$/Ag	5.9×10^{-7}	560	S5
**ITO//$PEA_2MA_{n-1}Pb_nI_{3n+1}$(n=5)/ PCBM/BCP/Ag**	**0.29**	**550**	**This Work**

Table S2 Summary of R_λ based on self-powered perovskite film

Photodetector Structure	Responsivity (A W^{-1})	Wavelength (nm)	Refs.
FTO/$MAPbCl_3$/Spiro-OMeTAD/Ag	0.005	398	S6
PET/ITO/C_{60}/perovskite/Spiro-OMeTAD/Ag/MoO_x/ITO	0.145	420	S7
ITO/PEDOT:PSS/$Cs_3Bi_2I_6Br_3$/C_{60}/BCP/Ag	0.015	400	S8
ITO/NiO_2/$CsPbCl_3$/C_{60}/BCP/Au	0.398	360	S9
FTO/PEDOT:PSS/ $MAPbI_3$/ETL/Al	0.25	540	S10
ITO/ZnO:PBI-H/$MAPbI_3$/Spiro-OMeTAD /Ag	0.35	670	S11
PEN/ITO/In_2S_3/$MAPbI_3$/Spiro-OMeTAD /Ag	0.45	473	S12
**ITO/PEDOT:PSS/$MAPbI_3$/$PC_{61}BM$/Bphen/Ag**	**0.57**	**760**	**This Work**

Table S3 Summary of R based on self-powered 2D and 3D perovskite film

Photodetector Structure	Light Intensity (W cm^{-2})	Responsivity (A W^{-1})	Laser wavelength (nm)	Refs.
Au/iBA$_2$(MA)$_{n-1}$PbnI$_{3n+1}$/Au	7×10^{-4}	0.333	532	S13
FTO/NiO$_x$/MAPbI$_3$/PCBM/PPDIN$_6$/Ag		0.0637	450	S14
ITO/PEDOT:PSS/MAPb(I$_{1-x}$Br$_x$)$_3$ /PCBM/Ag	6.36×10^{-3}	0.331	780	S15
ITO/(FAPbI$_3$)$_{0.97}$(MAPbBr$_3$)$_{0.03}$/Spiro-OMeTAD/Au	3.54×10^{-4}	0.053	254	S16
ITO/SnO$_2$/CsPbBr$_3$/PTAA/Au	6.41×10^{-2}	0.3	473	S17
ITO/PEALD-SnO$_2$/ CsPbBr$_3$MCs/PTAA/Au	6.4×10^{-4}	0.206	473	S18
ITO/SnO$_2$/MAPbI$_3$ MC/C	2.6×10^{-7}	0.26	473	S19
ITO/SnO$_2$/CsPbBr$_3$/MoO$_3$/Au	1.45×10^{-7}	0.091	473	S20
ITO/PEDOT:PSS/PEA$_2$ MA$_{n-1}$Pb$_n$I$_{3n+1}$(n=4)/PCBM/Bphen/Al	3.9×10^{-5}	0.46	600	S21
**ITO/PEDOT:PSS/FPEA$_2$MA$_{n-1}$Pb$_n$I$_{3n+1}$(n=5)/PCBM/BCP/Ag**	**1.5×10^{-7}**	**0.4**	**532**	**This Work**

Supplementary References

[S1] P. Gui, J. Li, X. Zheng, H. Wang, F. Yao et al., Self-driven all-inorganic perovskite microplatelet vertical Schottky junction photodetectors with a tunable spectral response. J. Mater. Chem. C **8**(20), 6804-6812 (2020). https://doi.org/10.1039/D0TC01473G

[S2] D Hao, D Liu, Y Shen, Q Shi, J Huang, Air-stable self-powered photodetectors based on lead-free CsBi$_3$I$_{10}$/SnO$_2$ heterojunction for weak light detection. Adv. Funct. Mater. **31**(21), 2100773 (2021). https://doi.org/10.1002/adfm.202100773

[S3] Y Pan, X Wang, Y Liao, Y Xu, Y Li, Epitaxial perovskite single-crystalline heterojunctions for filterFree ultra-narrowband detection with tunable spectral responses. ACS Appl. Mater. Interfaces **14**(44), 50331-50342 (2022). https://doi.org/10.1021/acsami.2c13126

[S4] E. Zheng, B. Yuh, G. A. Tosado, Q. Yu, Solution-processed visible-blind UV-A photodetectors based on CH$_3$NH$_3$PbCl$_3$ perovskite thin films. J. Mater. Chem. C **5**(15), 3796 (2017). https://doi.org/10.1039/C7TC00639J

[S5] A. A. Hussain, Constructing caesium-based lead-free perovskite photodetector enabling self-powered operation with extended spectral response. ACS Appl. Mater. Interfaces **12**(41), 46317-46329 (2020). https://doi.org/10.1021/acsami.0c14083

[S6] S. Liu, S. Jiao, H. Lu, Y. Nie, S. Gao et al., Polycrystalline perovskite $CH_3NH_3PbCl_3$/amorphous Ga_2O_3 hybrid structure for high-speed, low-dark current and self-powered UVA photodetector. J. Alloys Compd. **890**, 161827 (2020). https://doi.org/10.1016/j.jallcom.2021.161827

[S7] M. Zhang, Q. Lu, C. Wang, H. Dai, J. He et al., High-performance and stability bifacial flexible self-powered perovskite photodetector by surface plasmon resonance and hydrophobic treatments. Org. Electron. **99**, 106330 (2021). https://doi.org/10.1016/j.orgel.2021.106330

[S8] D. Liu, B.-B. Yu, M. Liao, Z. Jin, L. Zhou et al., Self-powered and broadband lead-free inorganic perovskite photodetector with high stability. ACS Appl. Mater. Interfaces **12**(27), 30530-30537 (2020). https://doi.org/10.1021/acsami.0c05636

[S9] X. Wu, J. Sun, H. Shao, Y. Zhai, L. Li et al., Self-powered UV photodetectors based on $CsPbCl_3$ nanowires enabled by the synergistic effect of acetate and lanthanide ion passivation. Chem. Eng. J. **426**, 131310 (2017). https://doi.org/10.1016/j.cej.2021.131310

[S10] J. Ghosh, G. Natu, P. Giri, Plasmonic hole-transport-layer enabled self-powered hybrid perovskite photodetector using a modified perovskite deposition method in ambient air. Org. Electron. **71**, 175-184 (2019). https://doi.org/10.1016/j.orgel.2019.05.021

[S11] Q. Zhang, M. Shou, Y. Xu, J. Zheng, X. Wen et al., Photo-switchable electron-transporting layers for self-driven perovskite photodetectors towards high detectivity. J. Mater. Chem. C **8**, 16506-16512 (2020). https://doi.org/10.1039/d0tc03649h

[S12] M. Wang, F. Cao, L. Meng, W. Tian, L. Li, High-performance flexible self-powered photodetector based on perovskite and low-temperature processed In_2S_3 nanoflake film. Adv. Mater. Interfaces **6**(4), 1801526 (2019). https://doi.org/10.1002/admi.201801526

[S13] Z. Lai, Y. Meng, Q. Zhu, F. Wang, X. Bu et al., High-Performance Flexible Self-powered photodetectors utilizing spontaneous electron and hole separation in quasi-2D halide perovskites. Small **17**(23), 2100442 (2021). https://doi.org/10.1002/smll.202100442

[S14] J. Wang, S. Xiao, W. Qian, K. Zhang, J. Yu et al., Self-driven perovskite narrowband photodetectors with tunable spectral responses. Adv. Mater. **33**(3), 2005557 (2021). https://doi.org/10.1002/adma.202005557

[S15] S. Qiao, Y. Liu, J. Liu, G. Fu, S. Wang, High-responsivity, fast, and self-powered narrowband perovskite heterojunction photodetectors with a tunable response range in the visible and near-infrared region. ACS Appl. Mater. Interfaces **13**(29), 34625-34636 (2021). https://doi.org/10.1021/acsami.1c09642

[S16] T. M. H.Nguyen, S. Kim, C. W. Bark, Solution-processed and self-powered photodetector in vertical architecture using mixed-halide perovskite for highly sensitive UVC detection. J. Mater. Chem. A **9**, 1269-1276 (2021). https://doi.org/10.1039/d0ta08738f

[S17] H. Zhou, Z. Song, C. R. Grice, C. Chen, J. Zhang et al., Self-powered $CsPbBr_3$ nanowire photodetector with a vertical structure. Nano Energy **53**, 880-886 (2018). https://doi.org/10.1016/j.nanoen.2018.09.040

[S18] H. Zhou, Z. Song, C. R. Grice, C. Chen, X. Yang, Pressure-assisted annealing strategy for high-performance self-powered all-inorganic perovskite microcrystal photodetectors. J. Phys. Chem. Lett. **9**(16), 4714-4719 (2018). https://doi.org/10.1021/acs.jpclett.8b01960

[S19] X. Pan, H. Zhou, R. Liu, D. Wu, Z. Song et al., Achieving a high-performance, self-powered, broadband perovskite photodetector employing $MAPbI_3$ microcrystal films. J. Mater. Chem. C **8**, 2028-2035 (2020). https://doi.org/10.1039/c9tc05668h

[S20] R. Wang, H. Zhou, B. Wu, D. Wu, L. Tao et al., Self-powered $CsPbBr_3$ perovskite nanonet photodetector with a hollow vertical structure. J. Phys. Chem. Lett. **12**(31), 7519-7525 (2021). https://doi.org/10.1021/acs.jpclett.1c02177

[S21] Y. Yan, Q. Wu, Y. Zhao, S. Chen, S. Hu et al., Air-stable and self-driven perovskite photodiodes with high on/off ratio and swift photoresponse. Small **14**(41), 1802764 (2018). https://doi.org/10.1002/smll.201802764
